# A Systematic Machine Learning Based Approach for the Diagnosis of Non-Alcoholic Fatty Liver Disease Risk and Progression

**DOI:** 10.1038/s41598-018-20166-x

**Published:** 2018-02-01

**Authors:** Sajida Perveen, Muhammad Shahbaz, Karim Keshavjee, Aziz Guergachi

**Affiliations:** 1 Department of Computer Science & Engineering, University of Engineering & Technology, Lahore, Pakistan; 2 Research Lab for Advanced System Modelling, Ryerson University, Toronto, ON M5B 2K3 Canada; 3 Dalla Lana School of Public Health, University of Toronto, Toronto, Ontario Canada; 4 Ted Rogers School of Information Technology Management, Ryerson University, Toronto, Ontario Canada; 5 Department of Mathematics & Statistics, York University, Toronto, Ontario Canada

## Abstract

Prevention and diagnosis of NAFLD is an ongoing area of interest in the healthcare community. Screening is complicated by the fact that the accuracy of noninvasive testing lacks specificity and sensitivity to make and stage the diagnosis. Currently no non-invasive ATP III criteria based prediction method is available to diagnose NAFLD risk. Firstly, the objective of this research is to develop machine learning based method in order to identify individuals at an increased risk of developing NAFLD using risk factors of ATP III clinical criteria updated in 2005 for Metabolic Syndrome (MetS). Secondly, to validate the relative ability of quantitative score defined by Italian Association for the Study of the Liver (IASF) and guideline explicitly defined for the Canadian population based on triglyceride thresholds to predict NAFLD risk. We proposed a Decision Tree based method to evaluate the risk of developing NAFLD and its progression in the Canadian population, using Electronic Medical Records (EMRs) by exploring novel risk factors for NAFLD. Our results show proposed method could potentially help physicians make more informed choices about their management of patients with NAFLD. Employing the proposed application in ordinary medical checkup is expected to lessen healthcare expenditures compared with administering additional complicated test.

## Introduction

NAFLD is a common clinico-pathologic entity that includes a wide spectrum of liver disorders. This ranges from simple steatosis (excessive fat accumulation in liver) to steatohepatitis (liver cell injury and inflammation), advanced fibrosis and rarely, progression to cirrhosis and hepatocellular carcinoma. It is marked by hepatic triglyceride (TRG) accumulation in liver parenchyma that adds to liver weight by at least 5%, however, it is not caused by consumption of alcohol^1,2^.

NAFLD prevalence is increasing rapidly. This increase is quite noteworthy in western countries. According to Souza *et al*.^3^ the prevalence of NAFLD is estimated at 45% in Hispanic-Americans, 33% in European-Americans and 24% in African-American. Other studies show that it can affect up to 30% of the general population^4^. Its relative prevalence is estimated to be 69% among individuals with type 2 diabetes mellitus/glucose intolerance^5^ when diagnosed by ultrasonography, 87% when diagnosed using biopsy or magnetic resonance imaging^6,7^. The literal pervasiveness of NAFLD still remains unidentified due to heterogeneity in diagnosis, the population under consideration and the degree of diversity across various factors associated therewith^8^.

Although the pathogenic mechanism of NAFLD is incompletely understood, the majority of NAFLD patients remains oblivious of their diagnosis until some major complications are encountered or it is diagnosed during tests carried out for some other reasons^9^. NAFLD bears bidirectional association with Metabolic Syndrome (MetS)^10^. MetS is a cluster of risk factors that significantly exposes an individual to coronary heart disease, diabetes mellitus, endocrine-metabolic diseases and chronic renal failure^7,11,12^. Hence a space is available to make use of these factors for diagnosis of NAFLD risks. In 2005, the clinical criteria Adult Treatment Panel III (ATP III) were updated by the National Heart Lung and Blood Institute (NHLBI) and the American Heart Association (AHA). According to ATP III, the MetS is diagnosed by the existence of three or more risk factors^6,13,14^ given in Table 1.

**Table 1:** Definition of the metabolic syndrome, according to recent classifications^13^.

Risk Factor/Features	National Cholesterol Education Program, ATP-III	International Diabetes Federation	Joint statement of IDF, NHLBI, AHA, WHF, IAS, IASO
Abdominal obesity, (waist circumference)	>102 cm (males), >88 cm (females)	≥94 cm (males), ≥80 cm (females)(ethnic differences)	≥94 cm (males), ≥80 cm (females)(ethnic differences)
Lipoprotein level	TRG ≥150 mg/dL or treated for dyslipidemia	TRG ≥150 mg/dL or treated for dyslipidemia	TRG ≥150 mg/dL or treated for dyslipidemia
HDL level	HDL-Chol <40 mg/dL (males); <50 mg/dL (females)	HDL-Chol <40 mg/dL (males); <50 mg/dL (females)	HDL-Chol <40 mg/dL (males); <50 mg/dL (females)
Blood pressure	≥130/85 mmHg or treated for Htx	≥130/85 mmHg or treated for Htx	≥130/85 mmHg or treated for Htx
Fasting Glucose (FG)	≥110 mg/dL or treated for DM	≥100 mg/dL or treated for DM	≥100 mg/dL or treated for DM
**Note**	3 of the above	Abdominal obesity + 2 of the above	3 of the above
**Associated Risk**	**Defining Factors**		
0	No abdominal adiposity and no other features of MetS		
1	Abdominal adiposity		
2	Abdominal adiposity +1 feature of MetS (i.e. atherogenic dyslipidemia, low HDL cholesterol and/or high TRG, hypertension or fasting hyperglycemia/glucose intolerance/diabetes)		
3	Abdominal adiposity + 2 features of MetS		
4	Abdominal adiposity + 3 features of MetS		

Quantitative score to estimate the impact of metabolic factors on nonalcoholic fatty liver disease^6^. FG, Fasting glucose, HDL, high-density lipoprotein, BMI, body mass index, DM, Diabetes Mellitus, TRG, Triglyceride, MetS, metabolic syndrome.

There are several methods in the literature individually to diagnosis diabetes, kidney or heart disease. Parthiban *et al*.^15^ proposed Naïve Bayes based method to diagnose heart disease using diabetic dataset that contain no prior information related to heart disease. However, there is no machine learning based method to identify NAFLD risk from diabetic dataset with no prior information related to NAFLD risk; using risk factors based on ATP III clinical criteria proposed in 2005 for metabolic syndrome to our knowledge.

Early stage detection and diagnosis of NAFLD risk is needed for a variety of reasons. If detected at an early stage and contained promptly, it may be possible to check NAFLD from getting worse and decrease the quantity of fat in liver effectively. About 50% of individuals with compensated cirrhosis owing to NAFLD would either require liver transplant or pass away due other disorders triggered by liver associated diseases^16^. NAFLD individuals demonstrate significantly higher premature mortality rate than the general population^17^. Identification of novel treatments is bound on the early and reliable identification of NAFLD risk.

Data mining has been of tremendous interest in healthcare community for some decades now, which identifies useful information by sifting through huge quantities of data using statistical as well as pattern recognition and mathematical techniques^18^. In this setting, EMRs demonstrate a vital role through cognizing of repetitive clinical measurements related to a patient’s condition over time along with vital signs, diagnosis, procedures, prescribed medications and demographics^19^. In principle this comprehensive information from each medical encounter can be incorporated to build models that take the semantics of such data into account, use information and knowledge intelligently and effectively help disease prediction as well as progression^18^. Hence it is needed to analyses the already available huge diabetic data sets to discover some incredible facts which may help in producing some prediction model.

To overcome the above-mentioned issues and provide for a rapid and detailed analysis of medical data the present study proposes a Decision Tree (DT) based prediction model to investigate the risk of developing NAFLD in the Canadian population using risk factors proposed for MetS by ATP III. It may be noted that the risk factors used in our proposed method are those that are put forward in Adult Treatment Panel III (ATP III) clinical criteria proposed in 2005 to diagnose metabolic syndrome and are not direct indicators of NAFLD.

## Methodology

### HealthCare data

The data used in this research is acquired from the Canadian Primary Care Sentinel Surveillance Network (CPCSSN) which is a pioneer multi-disease EMR-based surveillance system of Canada. Data from all participating networks, provided by family physicians and other primary care providers, are aggregated into a single national database (http://cpcssn.ca/). CPCSSN contains 667907 records for a period ranging from 2003 to Sept 30, 2013 and every record comprises of various attributes regarding vital signs, diagnosis and demographics. This dataset has previously been used by Mashayekhi *et al*.^19^ to assert the discriminability of the Framingham diabetes risk model in Canadian population. An abstract overview of CPCSSN dataset is given in Table 2.

**Table 2:** Characteristics of the population in the Canadian primary care sentinel surveillance network database.

Predictors	Findings
**Demographic (Gender, Age)**	
Male, sample size (%)	287964 (43)
Female, sample size (%)	379561, (57)
Male age mean ±SD,Years	47.2 ± 25.1
Female age mean ± SD,Years	49.5 ± 24.8
**Vital Signs/ clinical measures**	
Diastolic BP mean ± SD, mm Hg	73.3 ± 12.4
Systolic BP, mean ± SD, mm Hg	121.9 ± 16.9
Unknown disease frequency (%)	393344 (59)
COPD frequency (%)	15926 (2.4)
Dementia frequency (%)	12007 (1.8)
Depression frequency (%)	62682 (10)
Diabetes Mellitus frequency (%)	40637 (6)
Epilepsy frequency (%)	5553 (0.8)
Hypertension frequency (%)	88615 (13)
Osteoarthritis frequency (%)	47606 (7)
Parkinson’s Disease frequency (%)	1825 (0.2)
**Lab Values**	
FG, mean ± SD, mmol/L	5.54 ± 1.91
Triglycerides, mean ± SD, mmol/L	1.43 ± 1.21
HDL, sample size, mean ± SD, mmol/L	1.38 ± 0.41
BMI, mean ± SD, kg/m^2^	26.54 ± 7.37

SD, standard deviation; BP, Blood Pressure, BMI, body mass index, FG, Fasting glucose, HDL, high-density lipoprotein, COPD, chronic obstructive pulmonary disease.

*Some patients have more than 1 disease in the database.

The consolidation of healthcare information from healthcare centers and hospitals in CPCSSN is an on-going job; hence, not all the information related to risk factors considered for the NAFLD risk prediction are available for all individuals, thus restricting the size of data. At this stage the dataset on clinical measurements are partial, about 627,180 patients out of 667,907 do not bear information for all the factors that are considered in this research based on ATP III for the prediction of NAFLD. The records that contain information for all the related factors are considered in this research. Hence, the final research sample after preprocessing contains 40637 records that include approximately 59% women and 40% men.

An abstract detail of our study sample and 7 potentially relevant risk factors proposed by ATP (III) clinical criteria in 2005 for metabolic syndrome identification are used in the context of NAFLD as listed in Table 3. Those are systolic blood pressure, diastolic blood pressure, high density lipoprotein (HDL) triglycerides (TRG), body mass index (BMI), and fasting blood glucose (FG). Additional demographic variables age and sex are also included in this study. All the records for lab values mentioned above for each patient are recorded in mmol/L and demographic and clinical characteristics are described using mean ± standard deviation for continuous variables and categorical data are expressed as frequencies and percentages.

**Table 3:** Characteristics of study samples without random under-sampling and with random under-sampling with uniform class distribution.

Predictors	Findings	
	Without random under-sampling	With random under-sampling
**Demographic (Gender, Age)**		
Male, sample size	16631	473
Female, sample size	24006	527
Overall maximum age, Years	103	93
Overall minimum age, Years	9	19
Overall age mean ± SD,Years	61.2 ± 14.2	59.48 ± 12.74
**Vital Signs/clinical measures**		
Systolic blood pressure, mean (SD), mm Hg	125.5 ± 15.7	127.3 ± 15.403
Diastolic blood pressure mean (SD), mm Hg	75.4 ± 9.7	77.064 ± 10.243
**Lab Values**		
FG, mean ± SD, mmol/L	5.4 ± 1.2	5.783 ± 1.935
Triglycerides, mean ± SD, mmol/L	1.4 ± 1.2	1.5 ± 1.31
HDL, sample size, mean ± SD, mmol/L	1.4 ± 0.4	1.248 ± 0.399
BMI, mean ± SD, kg/m^2^	28.5 ± 6.1	30.618 ± 6.164

The CPCSSN has received ethics approval from the research ethics boards of all host universities for all participating networks and from the Health Canada Research Ethics Board. All participating CPCSSN sentinel primary care providers provided written informed consent for the collection and analysis of their EMR data. All data are fully anonymized, using the PARAT tool from Privacy Analytics (Ottawa, Canada). The University of Engineering & Technology research ethics board provided a waiver of ethics review for this study. All animal experimental procedures were conducted in compliance with the guidelines and regulations for the use and care of animals. All methods were carried out in accordance with relevant guidelines and regulations.

### Proposed method

The study goal is to facilitate health care professionals/physicians in investigation or prediction of the risk of developing NAFLD in an individual using risk factors put forward in ATP III clinical criteria that are not direct indicators of NAFLD. As a crucial understanding of various risk factors and pathogenic mechanism of NAFLD is compulsory for individualized prevention, management and advanced diagnostic strategies. Let *D* be the Dataset and $D=\{{{\rm{S}}}_{1},{S}_{2},\ldots .,{S}_{{\rm{n}}}\}$ where S_i_ represents record of a particular patient in *D* and ${\rm{n}}=1,2,3,\ldots ,\,40,637$ and each ${{\rm{s}}}_{{\rm{i}}}=\{{{\rm{A}}}_{{\rm{i}}1},{{\rm{A}}}_{{\rm{i}}2},\ldots .,{{\rm{A}}}_{{\rm{im}}}\}$ vector of risk factors of an instance in *D* and ${\rm{m}}=1,2,3,\ldots ,7$. Nevertheless, the dataset of risk factors do not contain any class label whereas the evaluation and prognosis criteria based on DT that is a supervised classification algorithm. Hence, it is crucial to have categorical attributes upon which the dataset can be classified.

For this purpose we have taken quantitative scores to evaluate the impact of metabolic factors on NAFLD defined by the Italian Association for the Study of the Liver (IASF) depicted in Table 1^6,13^ along with a guideline explicitly defined for the Canadian population based on triglyceride (TRG) level^20^. As it would be worth exploring whether these reference levels of TRG would affect the classification accuracy of the prediction model. So, these defined TRG levels are used as the reference value for determining of NAFLD risk. As a recent study revealed that the prevalence of NAFLD in individuals without metabolic syndrome was 6.1%^6,21^. Furthermore, for ease of understanding, we convert TRG into ordinal categories, as the TRG attribute holds a range of numeric values. The risk of developing NAFLD in each patient is categorized into four mutually exclusive and exhaustive classes (1) Desirable; (2) Borderline-high; (3) High; (4) Very-High based on the values of TRG. This categorization has also been done based on the fact that a TRG value of 5.6 or > 5.6 mmol/L is taken to be high TRG for Canadians^6^. These categorizations are not gender specific and are based on Canadian guidelines as depicted in equations (1), (2), (3) and (4).

#### Patient segmentation

Risk classification and face-print: Let $D=\{{{\rm{s}}}_{1},\,{{\rm{s}}}_{2},\,\ldots ,{{\rm{s}}}_{{\rm{n}}}\}$ where s_i_ is a training instance in *D* augmented with a class vector ${\rm{C}}=\{{{\rm{c}}}_{1},\,{{\rm{c}}}_{2},\,\ldots ,{{\rm{c}}}_{{\rm{k}}}\}$ where C can assume at most four values as mentioned above in which two (Desirable (*L*_*D*_) and Borderline-High (*L*_*BH*_)) point to stability whereas the remaining two (High (*L*_*H*_) and Very-High (*L*_*VH*_) point to instability and high risk for developing NAFLD and each ${{\rm{s}}}_{{\rm{i}}}=\{{{\rm{A}}}_{{\rm{i}}1},\,{{\rm{A}}}_{{\rm{i}}2},\,\ldots ,{{\rm{A}}}_{{\rm{im}}}\}$ vector of attributes of a training instance in *D* that contain systolic blood pressure, diastolic blood pressure, high density lipoprotein (HDL) triglycerides (TRG), body mass index (BMI), and fasting blood glucose (FG). Where the range of TRG of an individual *S*_*i*_ denoted by *R*_*TRG*(*Si*)_ and each *S*_*i*_ augmented with a class label based upon *R*_*TRG*_ and qualitative scoring criteria depicted in Table 1.

1 $$\forall \,S\in {\rm{D}}\,{\rm{and}}\,{C}_{Desirable}\in C\therefore \,{S}_{i}\in {C}_{Desiable}\iff {R}_{TRG(Si)} < {L}_{D}$$

2 $$\forall \,S\in {\rm{D}}\,{\rm{and}}\,{C}_{BorderlineHigh}\in C\therefore \,{S}_{i}\in {C}_{BorderlineHigh}\iff {L}_{D} < {R}_{TRG(Si)} < {L}_{BH}$$

3 $$\forall \,S\in {\rm{D}}\,{\rm{and}}\,{C}_{High}\in C\therefore \,{S}_{i}\in {C}_{High}\iff {L}_{BH} < {R}_{TRG(Si)} < {L}_{H}$$

4 $$\forall \,S\in {\rm{D}}\,{\rm{and}}\,{C}_{VeryHigh}\in C\therefore \,{S}_{i}\in {C}_{VeryHigh}\iff {L}_{H} < {R}_{TRG(Si)}\ge {L}_{VH}$$

where *L*_*D*_, *L*_*BH*_, *L*_*H*_, *L*_*VH*_ can hold values <1.7, 2 0.2, 5.6 and >5.6 mmol/L respectively^6,20^.

The association of a particular individual to one of the above mentioned categories can then be evaluated using the above devised procedure depicted in equations (1), (2), (3) and (4). After evaluation process this association is considered as class label. Table 4 shows the study sample distribution across different categories that include categories that include (1) Desirable; (2) Borderline-High; (3) High; (4) Very-High based on the values of TRG. Then the instances are again stored in the database with befitting output label.

**Table 4:** Study sample distribution among different ordinal categories.

Categories	NAFLD	
	N	%
Desirable	30332	74.6
Borderline-High	5105	12.6
High	5011	12.08
Very-High	189	0.661
Total	40637	100.0

### Method for balancing class distribution

Prediction models are often developed on class-imbalanced data this is especially true about healthcare informatics^22^. A dataset is said to be imbalanced if there are significantly more data points of one class and fewer occurrences of the other class: for example, data gathered from screening programs usually include few patients with the disease (minority class samples) and many healthy subjects (majority class samples). Such models tend to achieve poor predictive accuracy in the minority class^23^. In addition, lots of medical research involves dealing with rare, but important medical conditions/events or subject dropouts in the longitudinal study^24–27^. Dealing with imbalanced datasets entails approaches such as advanced and improved classification techniques or balancing classes in the training data (data preprocessing) before feeding the data as input to the data mining algorithm. The later technique is preferred as it has wider application and most widely used strategy to improve the predictive accuracy of the minority class.

The main strategy of balancing classes is to either increasing the frequency of the minority class or decreasing the frequency of the majority class. This is done to obtain approximately the same number of instances for both the classes in order to obtain a balanced distribution prior to building the prediction model. The data imbalance problem in our data is clearly shown in Table 4.

The study sample distribution is imbalanced among above mentioned ordinal categories ((1) Desirable; (2) Borderline-High; (3) High; (4) Very-High) as shown in Table 4. So, we adopted a random under-sampling method. Random Under-sampling aims to balance class distribution by randomly selecting majority class examples. This method is used when quantity of data is sufficient. By keeping all samples in the minority class and randomly selecting an equal number of samples in the majority class. This is done until the majority and minority class instances are balanced out, a balanced new dataset can be retrieved for further modeling. The dataset reduced to 936 records with balanced distribution for each class and an abstract detail in given in Table 3.

## Supervised machine learning

Since the aim of this research is to analyze the risk of developing NAFLD in an individual and to facilitate physician or decision maker to evaluate risk progression in each individual to make informed choices about their management and improve health condition along with reduce healthcare cost. After evaluating NAFLD risk, the next step is to determine the contribution of each factor in the onset of NAFLD as facts are crucial to comprehend the prognosis.

From the knowledge discovery perspective, the capability to track and assess each step in the process of decision-making is one of the most important and primary factors for relying on the decisions gained from data mining techniques^28^. Decision tree is one example of such methods that possess ability to communicate the results in a simple self-explanatory symbolic and visual format with satisfactory accuracy levels in various domains. It incorporates multiple predictors in a simple step by step manner, whose semantics are intuitively clear and easy to interpret for experts, as they can see the structure of decisions in the classifying process^28,29^. Different alternative even without complete information in term of risk and probable values can be compared. Although current state-of-the art classifiers (e.g. Support Vector Machines^22,30,31^) or ensembles of classifiers^32–34^ (e.g. Random Forest^35,36^) significantly outperform classical decision tree classification models in terms of classification accuracy or other classification performance metrics, but not suitable for knowledge discovery process.

**Figure 1:**
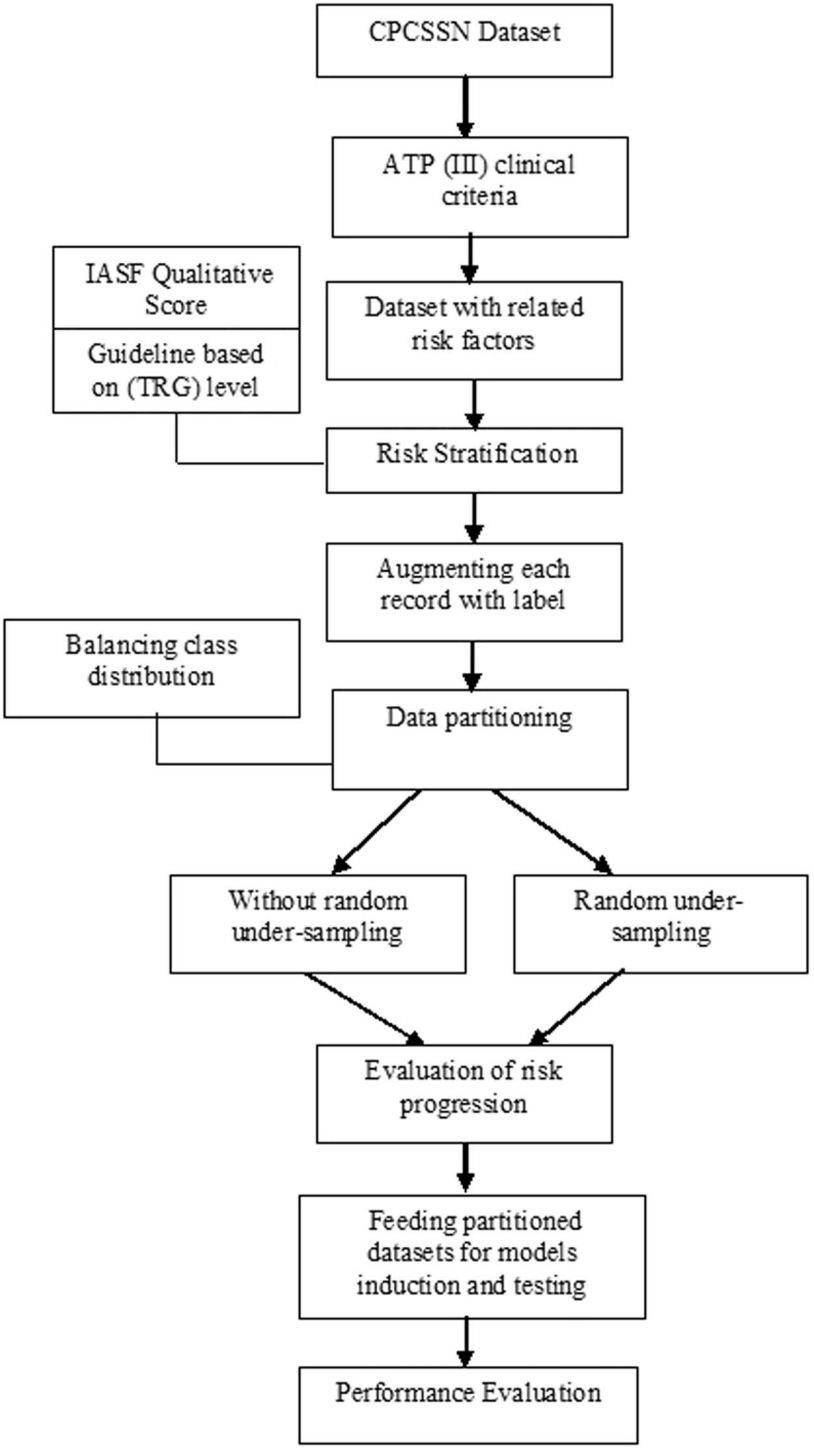
Abstract overview of proposed methodology.

Therefore, the present study rationally involves J48 DT (C4.5) a promising technique for predictive modeling^37^. Early stage prediction of risk for developing NAFLD is not sufficient; a physician or decision maker may also want to know the causes for developing NAFLD risk. The DT maps all risk factors rules to facilitate physician or decision maker to address each individual risk factor to make informed choices about their management. The resulting information may be useful for making interventions to halt or delay NAFLD onset. An abstract Over view can be seen in Fig. 1.

### Decision tree classification

Classification is a procedure of building a model of class attributes from a dataset, to assign a class label to previously unseen record as accurately as possible. DT is a supervised classification model aimed at partitioning data into homogeneous groups in term of variables to be predicted using entropy. If the partition of data is completely homogeneous, entropy will be zero. Entropy is a gauge to measure the level of disorder in data. Basically, it defines the quantity of information provided by an event. The lower the entropy of an event is (it is rare), the higher the information it provides. Information gain is based on decrease in entropy^37^. DT is a tree like hierarchical structure that consists of branches (arcs) and three types of nodes, root, intermediate and leaf node respectively that correspond to the sequence of decision rules.

The attribute that divides the data efficiently is selected as a root node. Next, a child node is selected by calculating Information Gain or some other statistical measure. The branches coming from an internal node are labeled with values of the attribute that a particular node can assume and each branch from root to leaf node represent an if-then rule for the prediction of class for a newly seen instance. Decision trees are reasonable to build, easy to perceive and integrate with database systems^38,39^. Several measures for optimal attribute selection are have been identified in the literature, such as gini index in CART, information gain in ID3 and gain ratio C4.5^40^. Let $D=\{{{\rm{s}}}_{1},\,{{\rm{s}}}_{2},\,\ldots ,{{\rm{s}}}_{{\rm{n}}}\}$ where s_i_ is a training instance in *D* augmented with a class vector ${\rm{C}}=\{{{\rm{c}}}_{1},\,{{\rm{c}}}_{2},\,\ldots ,{{\rm{c}}}_{{\rm{k}}}\}$ and each ${{\rm{s}}}_{{\rm{i}}}=\{{{\rm{A}}}_{{\rm{i}}1},\,{{\rm{A}}}_{{\rm{i}}2},\,\ldots ,{{\rm{A}}}_{{\rm{im}}}\}$ vector of attributes of a training instance in *D*. The following equations are used to measure the entropy and information gain.

5 $$Entropy(D)=-\sum _{i=1}^{n}{{\rm{p}}}_{{\rm{i}}}{\mathrm{log}}_{2}{{\rm{p}}}_{{\rm{i}}}$$

where p_i_ is the portion of data belonging to a particular Class and p_i_ = s_i_/c_i_. Given a set D of objects, and an attribute A,

6 $${\rm{Information}}\,\_{\rm{Gain}}({\rm{D}},{\rm{A}})=Entropy(D)\,\sum _{j=1}^{l}({{\rm{p}}}_{{\rm{j}}}\,\times \,Entropy({{\rm{p}}}_{{\rm{j}}}))$$

where values (p_j_) is the set of all possible values for attribute A and *j* can be upto 1, 2, ..., *l*.

#### J48 decision tree

J48 is an open source java implementation of C4.5 algorithm in WEKA; primarily an extension of ID3 developed by Quinlan in 1986^41^. It is a variation of information gain, generally used to overcome the effect of biasness. An attribute with the highest gain ratio is selected in order to build tree as a splitting attribute^37^. Gain ratio based DT outperforms than information gain in terms of both accuracy and dealing complex tasks^11,38,42,43^. Gain ratio, is defined as follows:

7 $$Gai{n}_{Ratio(D,A)}=\frac{Entropy(D){\sum }_{j=1}^{l}({{\rm{p}}}_{{\rm{j}}}\times Entropy({{\rm{p}}}_{{\rm{j}}}))}{Splitin{g}_{Info}}$$

**Figure 2:**
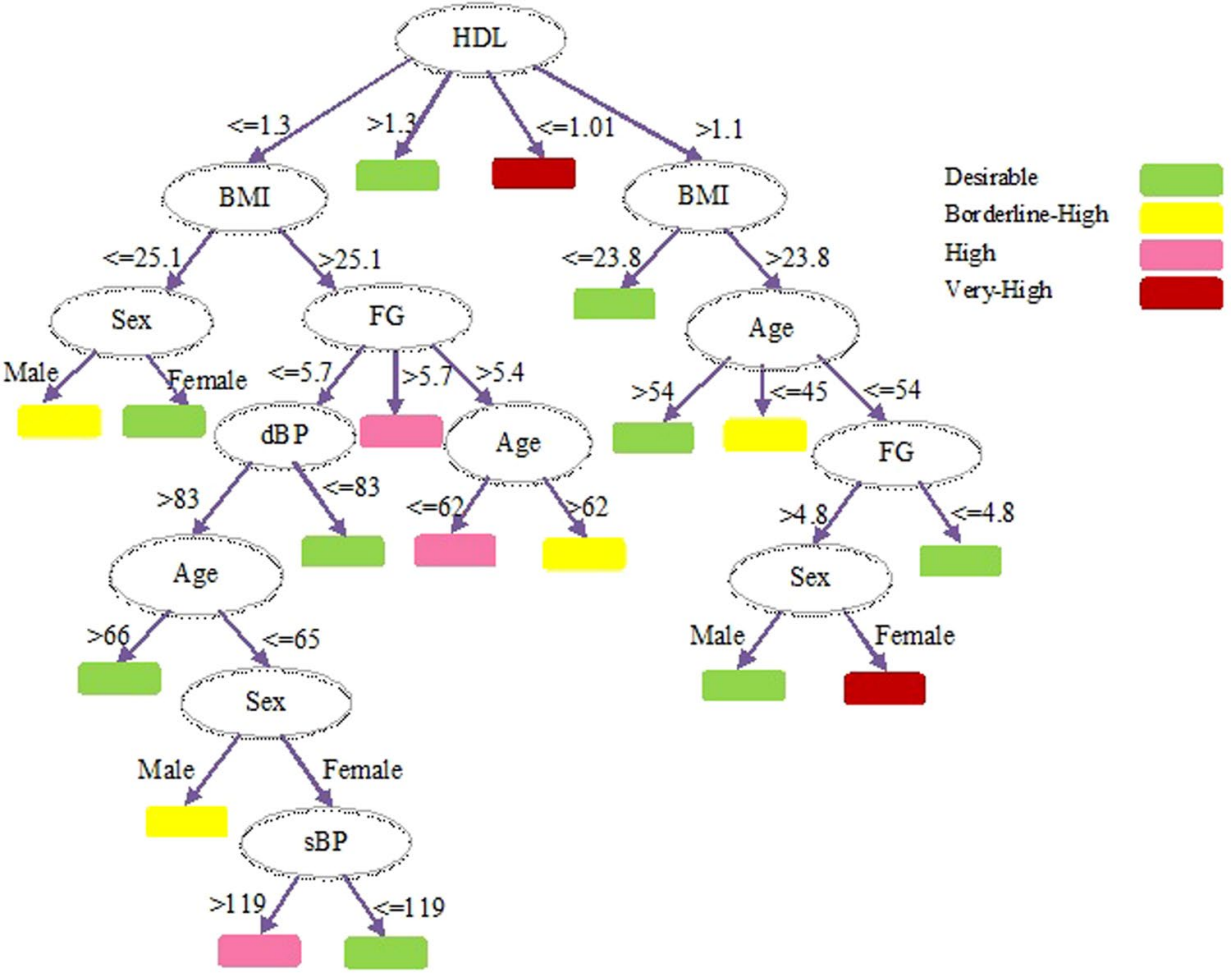
Decision tree drawn from CPCSSN Dataset.

The experiments were run with following settings: The confidence factor that represents a threshold value of allowed inherent error in data (whether an attribute is inside the confidence interval of the assigned class) while pruning the decision is set to 0.5 along with Subtree raising pruning. The minimum number of instances at a single leaf node for which confidence interval is computed was set to 20 in order to obtain simpler and smaller decision trees. Binary split is set to false basically this selection criteria control the visual outlook of the tree. The developed decision tree is shown as Fig. 2.

### Performance metrics

The level of effectiveness of the classification methods can be distinguished with number of correct and incorrect classifications in each possible case of the variables being classified. Given a particular classification algorithm and a data instance, there exist four possibilities. If the instance is positive and it is classified as positive, it is counted as a true positive (equation 8); if it is classified as negative, it is counted as a false negative. If the instance is negative and it is classified as negative, it is counted as a true negative; if it is classified as positive, it is counted as a false positive (equation 9).

8 $$TP=\frac{Correctly\,classified\,positives\,instances\,}{Total\,no.of\,positives}$$

9 $$FP=\frac{Incorrectly\,classified\,negative\,instances}{Total\,no.of\,negative\,instances}$$

To assess the discriminative capability of J48 classifier in both datasets as described above most frequently used performance measures such as Micro- and Macro-average of Precision, Recall and F-measure, Matthews Correlation Coefficient (MCC) and Area under Receiver Operating Characteristic (AROC) curves are incorporated as a tool. These are straightforward and well accepted comparison measure for multi class classifier^11,30,44–46^. Following formulas are used to measure above mentioned performance measures are shown below.

10 $$Precisio{n}_{\mu }=\frac{{\sum }_{n=1}^{l}T{P}_{n}}{{\sum }_{n=1}^{l}TP+FP}\quad Precisio{n}_{M}=\frac{{\sum }_{n=1}^{l}\frac{T{P}_{n}}{T{P}_{n}+F{P}_{n}}}{l}$$

11 $$Recal{l}_{\mu }=\frac{{\sum }_{n=1}^{l}T{P}_{n}}{{\sum }_{n=1}^{l}T{P}_{n}+F{N}_{n}}\quad Recal{l}_{M}=\frac{{\sum }_{n=1}^{l}\frac{T{P}_{n}}{T{P}_{n}+F{N}_{n}}}{l}$$

12 $$F-Measur{e}_{\mu }=\frac{2}{\frac{1}{Precisio{n}_{\mu }}+\frac{1}{Recal{l}_{\mu }}}$$

13 $$MCC=\frac{(TP\times TN-FP\times FN)}{\sqrt{(TP+FP)(TP+FN)(TN+FP)(TN+FN)}}$$

where *l* is the number of classes, *TP* true positives instances, *TN*, true negative, *FP* the number of false positives and *FN* the number of false negatives.

MCC performance measure to evaluate the performance of our proposed model. In the most general case, MCC is a good compromise among discriminancy, consistency and coherent behaviors with imbalanced class distribution as in our case (see Table 4) and randomization. It is in essence an association between the observed and predicted binary classifications; ranges between −1 and + 1. Where −1 depicts a perfect inverse prediction between prediction and observation and a coefficient of + 1 represents a perfect prediction, 0 no better than random prediction. MCC correlation coefficient value is calculated from confusion matrix for each class ((1) Desirable, (2) Borderline-High, (3) High, (4) Very-High).

We also incorporated the AROC curve for performance evaluation. It fundamentally characterizes by the amalgamation of sensitivity and specificity for individual possible cutoff value of the non-discrete test result that can be considered to express positive and negative test results. Theoretically, the AROC can have values ranges from 0 to 1, whereas a classifier with best discrimination capability will take the value of 1. Nevertheless, the practical lower bound for classification with random discrimination capability is 0.5 which indicate the classifier with no discriminative capability. Whereas classifiers that have AROC value significantly higher than 0.5 indicates that it has at least some power to discriminate. Supplementary notations related to AROC curves are.

14 $${\rm{S}}ensitivity=Recall$$

15 $$Specificity=\frac{TN}{FP+TN}$$

## Results

The multiclass labeled dataset of 7 risk factors for 40,637 individuals over a period of 10 years is incorporated in this study. The degree of distribution of each class is given in Table 4. We incorporated both balanced and unbalanced datasets in order to obtain a better insight on for which settings the proposed technique contributes to the classic DT. Hold out method is adopted for model building. Both datasets are further divided into two subsets for training and testing 66% and 34% respectively. An abstract detail of each study sample is presented in Table 3. Both datasets are then fed and mapped onto a decision tree using J48 (C4.5) algorithm in WEKA (3.8 Version). The experimental results obtained from both unbalanced and balanced datasets are presented in Tables 5 and 6 respectively.

**Table 5:** Detailed performance analysis of prediction model without random under-sampling.

	Class				
	Desirable	Borderline_high	High	Very_High	Weighted Avg.
TP Rate	0.937	0.05	0.296	0.024	0.762
FP Rate	0.777	0.03	0.053	0.001	0.542
Precision_µ_	0.78	0.493	0.573	0.133	0.669
Recall_µ_	0.937	0.451	0.396	0.024	0.735
F-Measure_µ_	0.851	0.279	0.349	0.04	0.676
Precision_M_	0.757	0.561	0.651	0.416	0.677
Recall_M_	0.832	0.59	0.503	0.366	0.713
MCC	0.328	0.247	0.195	0.055	0.299
AROC	0.748	0.631	0.738	0.507	0.731

**Table 6:** Detailed performance analysis of prediction model with random under-sampling.

	Class				
	Desirable	Borderline-High	High	Very_High	Weighted Avg.
TP Rate	0.574	0.53	0.511	0.637	0.582
FP Rate	0.108	0.197	0.374	0.11	0.223
Precision_µ_	0.62	0.587	0.468	0.647	0.594
Recall_µ_	0.516	0.654	0.672	0.687	0.637
F-Measure_µ_	0.592	0.669	0.504	0.598	0.614
Precision_M_	0.646	0.603	0.581	0.597	0.610
Recall_M_	0.547	0.678	0.72	0.667	0.661
MCC	0.204	0.377	0.167	0.164	0.276
AROC	0.748	0.812	0.693	0.809	0.746

The proposed method was able to classify 76% of the input instances correctly without random under-sampling. To evaluate the overall discriminative capability of multivariate classifier in Canadian healthcare data without random under-sampling different performance measure are used a tool. It exhibited a precision_µ_ of 66%, recall_µ_ of 73%, F-measure_µ_ of 67%, and AROC 73% on average, showing a fairly significant discriminative capability. The results for the all the cases show that MCC range from 0.055 to 0.328.

### Random under-sampling results

In order to incorporate balance distribution among ordinal classes under-sampling is applied on CPCSSN database. By keeping all samples in the minority class and randomly selecting an equal number of samples in the majority class. This is done until the majority and minority class instances are balanced out, a balanced new dataset can be retrieved for further modeling. The dataset reduced to approximately 939 records with balanced distribution for each class and an abstract detail in given in Table 3. In this case we have taken approximately 250 samples without replacement from each class and combined them with minority class (very-high). The balanced dataset is further divided into two subset training and test to build and validate the prediction model. The classifier, experimental settings and required parameters values for model building are explicitly mentioned in the method section.

To evaluate the overall discriminative capability of multivariate classifier different accuracy measures are used. Table 6 lists the results. Specificity enhanced compared to that without random under-sampling. In contrast, slight variation observed in the AROCs. The results btained from balanced dataset with random under-sampling exhibited a precision_µ_ of 59%, recall_µ_ of 63%, F-measure_µ_ of 61%, AROC 74% on average showing a fairly significant discriminative capability and MCC range from 0.164 to 0.377.

## Discussion

As mentioned earlier NAFLD is associated with metabolic disturbances and both are bi-directionally associated. It is a very complex clinical condition with different etiology involving a multitude of physiological mechanisms and symptoms^14^. Selecting potentially relevant data is crucial for building an efficient model from EMRs. Therefore, the major clinical factors considered in the ATP III clinical criteria for MetS are incorporated in the context of NAFLD as a basis for early stage screening of individuals at risk for developing NAFLD. Diabetes mellitus, NAFLD and metabolic syndrome frequently co-exist as they potentially share common risk factors of, imbalanced triglycerides and insulin resistance^47^.

We have taken quantitative scores defined by the Italian Association for the Study of the Liver (IASF) depicted in Table 1^6,13^, along with a guideline explicitly defined for the Canadian population based on triglyceride (TRG) level^20^ to evaluate the impact of metabolic factors on NAFLD risk; defined in equations (1), (2), (3) and (4).

Tomizawa *et al*.^48^ performed multivariate regression analysis to evaluate the efficiency of various risk factors in the prediction of NAFLD. These factors include TRG, HDL, low-density lipoprotein cholesterol (LDL), blood glucose (BG) and hemoglobin A1c (HbA1c). Experimental results demonstrate that TGR was the parameter most significantly associated with NAFLD (χ^2^ = 9.89, *P* = 0.0017) and also highlight that TRG is an elevated marker of NAFLD. A recent study also revealed that prevalence of NAFLD in individuals without metabolic syndrome was 6.1%^6^. So, in this research we have taken quantitative scores defined by IASF along with a guideline explicitly defined for the Canadian population based on triglyceride (TRG) level^20^. These defined levels are used as the reference value for determining of NAFLD risk. This was the first step in the development of NAFLD risk prediction model.

Early stage prediction of risk for developing NAFLD is not sufficient; a physician or decision maker may also want to know the causes for developing NAFLD risk. DT is one of the machine learning techniques possess ability to communicate the results in a simple self-explanatory symbolic and visual format with satisfactory accuracy levels in various domains. It incorporates multiple predictors in a simple step by step manner, whose semantics are intuitively clear and easy to interpret for experts, as they can see the structure of decisions in the classifying process^28,29^.

Hence, we evaluated J48 decision tree algorithm to identify contributing factors in the onset of NAFLD as facts are crucial to comprehend the prognosis. The most promising attribute with maximum information gain in our case HDL is selected as root. The root node is evaluated first when assessing NAFLD risk in an individual. If the range of HDL ≥ 1.3 the risk would be desirable that represent stability otherwise second node (BMI) with second highest information gain would be tested and this procedure continue until an instance is classified into one of the predefined categories mentioned above.

If we consider above rules, these rules are also valid according to medical perspective, as the analysis of NAFLD risk can also be done by the low HDL, high triglyceride and impaired FG^21,40,49^. Considering the cutoff value of HDL ≥ 1.3, that is supported by previous studies for desirable risk level^6,14,43^. Considering the second rule depicted in decision tree is also valid, latest research have depicted significant relation between low HDL, central obesity and the risk of developing NAFLD and/or MetS^3,6,21^. The IDF and ATP III also define MetS as the manifestation of central obesity, along with any two of the following factors. (1) Increased TRG level, (2) Low HDL, (3) hypertension (Systolic BP ≥ 130 or Diastolic BP ≥ 85 mmHg), (4) FPG ≥ 100 mg/dL, or earlier diagnosed as diabetic). Furthermore, an interesting fact described in existing studies can also be extracted from the above mentioned decision tree that the prevalence of NAFLD is higher in men with an “inverted U shaped curve”. It increases from young to middle-aged individual and declines in the elderly^6^, whereas increases with age in women^50^.

We also analyzed the performance of the predictive model on both with and without random under-sampling datasets taken from CPCSSN data. The AROC value reveals that the performance of the model on without random under-sampling data is 0.731 on average, as shown in Table 5. Given the 40,637 individuals records enrolled in CPCSSN over a period of 10 years, we can also predict the occurrence of at least 4562 NAFLD incidents correctly. A large cohort study revealed that NAFLD is correlated with 26% higher 5-year overall healthcare expenditures^51^. Thus limiting the economic burden of 4428 NAFLD patients.

Ordinarily, ultrasonography of abdomen is used to monitor the patients of NAFLD. Ultrasonography of abdomen test cost $150–$390 USD in the payment system for medical services in Canada if all individuals who underwent checkups are so tested, the total healthcare expenditure would rise by approximately 6,095,550 USD. Moreover, a significant large portion of these individuals would potentially be saved if individuals at high risk for developing NAFLD managed appropriately.

Furthermore, Table 6 demonstrates only a small variation in AROC using under-sampling. It did not increase the discriminability of predictive model and failed to incorporate informative records from the dataset. The AROC value of the predictive model depicts 0.746 discriminative ability of the classification using under-sampling. Some existing research have successfully applied under-sampling in predictive modeling^15,23^ however, the current research do not support their findings. Under-sampling techniques those consider informative records from data are worth examining to improve predictive capability.

The present research has two major limitations. Firstly, the research is carried out mainly on Canadian population, caution is required in TRG guidelines incorporation as the reference value for determining NAFLD risk and results generalization when dealing with other population. Secondly, we employed J48 decision tree algorithm for building prediction model, and did not incorporate any other classification algorithm. Further advanced research on the effectiveness of other methods is advised.

## Conclusion

Application of Data mining in analyzing the Electronic Medical Records is an efficient approach for discovering the existing relationships among variables that is ordinarily difficult to detect. From our proposed method we have shown that it can be exploited to extract implicit, useful, nontrivial associations even from factors that are not direct or explicit indicators of the class we are trying to predict. In this research we predicted the risk of developing NAFLD in an individual by incorporating noninvasive markers and gold standard machine learning method. The rationale behind our approach is divided in two parts: firstly relevant risk factors selection using ATP III clinical criteria proposed in 2005 for MetS and allocation of class label with respect to triglycerides (TRG) level along with qualitative scoring criteria proposed by IASF for extracting knowledge from the input data and evaluating the NAFLD risk in an individual. Secondly rule based reasoning and visualization of predictive results that can be employed in better understanding of the phenomena involving a multitude of physiological mechanisms and symptoms. The results demonstrate that the proposed technique is suitable with optimal discrimination for the assessment of NAFLD risk, understanding the contributing factors, producing accurate, specific and decision oriented rules to facilitate physician and make informed choices about their management and improve health condition. This can be extended to predict other type of ailments which arise from metabolic syndrome.
